# A novel path towards limiting non-exhaust particulate matter emissions of a commercial friction material through the addition of metallurgical slag

**DOI:** 10.1038/s41598-023-27932-6

**Published:** 2023-01-12

**Authors:** Priyadarshini Jayashree, Vlastimil Matějka, Mara Leonardi, Giovanni Straffelini

**Affiliations:** 1 Department of Industrial Engineering, University of Trento, Via Sommarive 9, Trento, Italy; 2 Department of Chemical and Physico-Chemical Processes, VSB – Technical University of Ostrava, 17. Listopadu 2172/15, 708 33 Ostrava, Czech Republic; 3 Brembo S.P.A., Stezzano, Bergamo, Italy

**Keywords:** Environmental sciences, Engineering, Materials science

## Abstract

Keeping recycling and the circular economy in mind, this study explores the reduction in emission of a highly optimized, commercially employed friction material formulation through the addition of metallurgical slags from a basic oxygen furnace in varying quantities from 6 to 38 wt%. The various compositions were paired with a pearlitic grey cast iron counterface and tested on a pin on disc tribometer. The friction coefficient and pin wear increased with the slag addition but were still within the permissible limit when compared to the original formulation. Specimens with higher slag content observed extremely compacted and extended secondary contact plateaus, which also recorded significant slag presence. The extended plateaus detached in the form of chunks from the mating surfaces, which settled on the equipment hardware and restricted the production of airborne particles. From an industrial symbiosis perspective, the addition of metallurgical slags proved to be a promising way of obtaining green friction materials with reduced emissions.

## Introduction

Friction materials for automotive braking systems must satisfy a vast array of requirements which includes stable and desirable friction coefficient (CoF), low system wear rate, and low noise and vibrations^1–3^. Therefore, friction material formulations include a range of constituents, sometimes more than 30 ingredients, which are divided into the binder, reinforcement, fillers, and friction modifiers (further divided into abrasive and lubricants)^4^. Recently, several efforts have been initiated to produce eco-friendly friction materials. New formulations are under development employing different types of wastes, including agricultural wastes and industrial wastes. This is done within the Industrial Symbiosis concept, i.e., in a process by which by-products or wastes from an *industry* or an *industrial process* become raw materials for another industry or process. This concept results in the more sustainable utilization of materials and helps in the development of a circular economy, which is the goal of the ‘European Commission’s Circular Economy Action Plan’^5–7^. Through the circular economy, the environmental footprints of industries are reduced, requirements for virgin materials are limited, and the need for landfill disposal is restricted, resulting in the value addition of the wastes^8–10^. From an environmental viewpoint, the material formulations must also satisfy the need of reducing the emission of polluting particulate matter (PM). In fact, among non-exhaust origin sources, the braking process currently contributes to 55 wt.% of PM_10_ fraction and to 21% of PM_2.5_ fraction (airborne particles having an aerodynamic diameter less than 10 µm and 2.5 µm respectively)^11,12^. PM emissions have been observed to have numerous health and environmental-related repercussions. They can contribute to respiratory and cardiovascular diseases, and a few of the PMs are classified as carcinogenic agents^13,14^.

In the present work, we investigated the possible use of metallurgical slags disposed of after reaction in the basic oxygen furnace (BOF) as an ingredient in a commercial friction material formulation. The slag typically constitutes CaO, MgO, silica, and iron-containing compounds and may also contain calcium and silicon-containing compounds^15^. The oxides are expected to behave as abrasives^16^. Additionally, it is a well-established observation that the secondary contact plateaus deposited on the mated surfaces during braking are predominantly made of Fe oxides^17,18^. The presence of Fe oxides through slags in a wear system could lead to interesting and positive results on the secondary plateau characteristics (which determines the friction, wear, and emission behavior of a system). The investigation of the emission and wear characteristics of the formulations were performed at 6, 12, 24, 32, and 38 wt.% of slag content, along with their wear debris characterization. The objective of the work is the development of an eco-friendly friction material with reduced emissions through an industrial symbiosis perspective. The novelty of the study is to significantly reduced PM emissions in a viable friction material formulation through the addition of slags, while also rigorously working towards reduction in the utilization of virgin raw materials.

## Research context

Various research projects have been undertaken to develop friction material formulations for automotive braking applications, wherein, different types of wastes are being employed—agricultural wastes and industrial wastes. For instance, Gomes Nogueira et al.^19^ and Gehlen et al.^20^ have conducted detailed studies on the friction and emission behavior of friction material formulations with the addition of untreated and heat-treated sieved rice husk. The waste effectively contributed towards the formation of well-compacted, smooth, and extended secondary contact plateaus, which in turn reduced the system wear and emissions. Ikpambese et al.^21^ added palm kernel fibers (PKF) in an asbestos-free automotive friction material composition. Results showed feasible friction, wear, and mechanical properties with the addition of 10 wt% PKF additions. Lastly, Ibrahim^22^ evaluated the addition of dry leaves from mango trees in a polymer matrix composite (typical resin used as a binder in a friction material formulation). The waste behaved as a solid lubricant, reducing the friction coefficient and wear rate to a great extent. The modification of friction material formulation with industrial wastes is also being studied. Gangwar et al.^23^ conducted a critical review on the tribological properties of friction material formulation with the addition of red mud, marble dust, and fly ash waste. It was seen that these wastes behaved as abrasives, providing superior thermal properties, permissible friction coefficient, and low wear rate. It was also shown that these materials were easily available, had low cost, and had the necessary density. Additionally, the combination of red mud and fly ash also fetches the required friction and wear magnitudes when compared to conventional formulation^24^. Mutlu et al.^25^ and Singh et al.^26^ added waste tire dust and cement kiln dust in different typical friction material formulations respectively. It was seen that the waste tire dust provided permissible wear characteristics and the combination of cement kiln dust with the phenolic resin binder assisted in enhanced friction performance. Wastes generated during the aluminum anodizing process were also investigated in different types of friction material formulations. At 12 wt% waste content, the friction, wear, and emission characteristics were similar to the virgin friction material composition^18^.

Perhaps, one of the most extensively tested industrial waste is metallurgical slags. Depending on different metallurgical processes, different kinds of slags are produced and are classified as ferrous, non-ferrous, and incineration slags, and are used in various applications like road construction and fertilizers^15^. Lately, some attempts were made to incorporate slags into friction material formulations. Wang et al.^27^ performed tribological, mechanical, and thermal behavior analysis on formulation made of polymer matrix composites at different slag content. The study observed superior wear resistance and stable CoF magnitudes and curves at different testing temperatures. Similarly, Erdogan et al.^28^ showed that the addition of slags in epoxy matrix composites at different loading conditions produced exemplary wear resistance at increased loading. Ozturk et al.^16^ and Jabbar et al.^29^ have reported that silica, MgO, and CaO behave as abrasives, implying that the BOF slag may potentially behave as an abrasive. Abrasives are known to elevate and stabilize CoF and remove the carbonaceous layer deposited on the mated surfaces. Majority of studies conducted on slag is based on its contribution towards friction and wear trends of the friction material formulation. However, no information is provided with respect to the emission/particle concentration trends and the analysis of the corresponding wear debris. Furthermore, the slag studies are conducted on laboratory prepared/formulated friction material formulation.

The benefits resulting from a proper re-utilization of metallurgical slags in friction materials for brake systems are in principle very relevant and pivotal. It has been reported that for an entire lifetime of a car, 16 pairs of brake pads are required, and since 2010, more than 70 million commercial vehicles (including cars) are manufactured every year^30^. It has also been reported that an estimated one billion cars are currently operating around the world. The growing demand for new materials for each batch of brake pads manufactured contributes to the depletion of resources and the production of by-products or wastes of a large magnitude.

## Methods

### Materials

**Figure 1:**
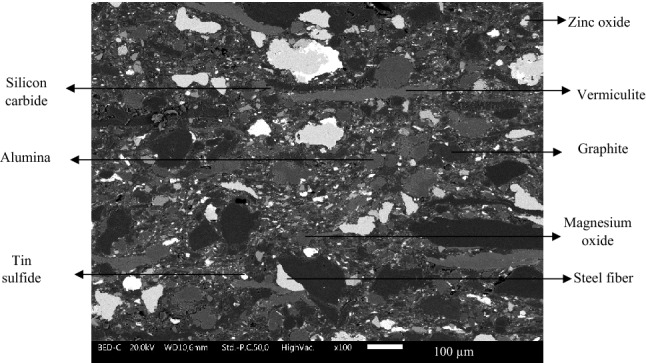
BSE SEM image showing the CFM specimen cross section with few marked constituents.

In this study, a commercial friction material formulation (named CFM), provided by Brembo Italy, was subjected to friction, wear, and emission analysis after the addition of a BOF slag. The CFM was selected for this analysis as it is an extensively tested and optimized formulation which is being used commercially for quite some time. From a preliminary XRD analysis, the main slag constituents were found to be iron oxide (FeO), followed by mullite, monticellite, silica, magnetite, and α- Fe. The complete composition of CFM was kept confidential. However, few of the constituents and the corresponding content in the formulation are shown in Table 1. Figure 1 shows the cross-section of a specimen made of CFM with few constituents highlighted.

**Table 1:** Few constituents of the CFM with their respective function in (wt%)^18^.

Constituents	Function	Content
Phenolic resin	Binder	8
Steel	Reinforcing fibers	30
Vermiculite, others	Fillers	24
Silicon carbide, magnesium oxide, aluminum oxide	Abrasives	25
Graphite, Tin sulfide, Zinc oxide	Lubricants	13

The CFM was modified by adding metallurgical BOF slag (named FS) to it. The virgin CFM was compared to the CFM compositions containing 6, 12, 24, 32, and 38 wt% of FS slag content. All formulations were tested in the form of pins. The pins were produced in house. The CFM and relevant slag contents were mixed thoroughly in a TURBULA® mixture for 30 continuous minutes. Next, the mixtures were subjected to a hot press procedure on a BUEHLER® Pneumet I equipment. The powders were tap pressed in a tool steel cylindrical mold at 150 °C, a pressure of 100 MPa, and a holding time of 10 min. Lastly, the green body specimens were subjected to curing at 200 °C for 4 h in a muffle urnace. After the completion of the production process, the average height and diameter of the pins were 10 mm^4,18,31^. The pins were paired with a counterface in the form of discs. The discs were made of pearlitic grey cast iron with an average diameter and thickness of 60 mm and 6 mm respectively. The properties and composition of the counterface is shown in Table 2.

**Table 2:** Composition and properties of the pearlitic grey cast iron counterface^18^.

Disc	Chemical composition, wt%							Hardness [HV 30]	Thermal Conductivity (W/mK)	Specific Heat (J/gK)
	C	Mn	Si	Sn	P	S	Fe			
Pearlitic grey cast iron	3.40	0.50	2.00	0.11	0.15	0.05	Rest	245 ± 6	52	0.447

### Testing methods and conditions

**Figure 2:**
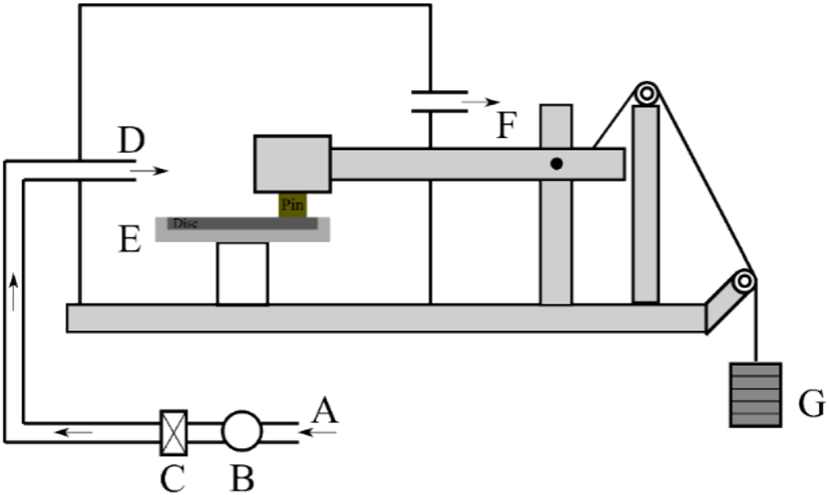
Pin on disc testing apparatus setup (A) Ambient air, (B) Fan, (C) HEPA filter, (D) Air introduced in the chamber, (E) Disc/Counterface, (F) Air outlet to the OPS, (G) Weights.

The dry sliding friction, wear, and emission analysis on the pin and disc pairings were conducted on a pin on the disc tribometer (PoD, Ducom Instruments). All trials were conducted on a new disc and at room temperature testing conditions. The testing conditions employed were a contact pressure of 1 MPa (79 N) and a constant sliding velocity of 1.51 m/s (600 rpm for a wear track of 48 mm). The testing parameters selected here replicate mild braking condition and was selected as this scenario would not only assist in evaluating the friction and wear behavior, but also provide information on the secondary contact plateau characteristics (extension, compaction, smoothness)^4,18^. The trials with each composition were repeated three times to obtain repeatability in the results. The PoD testing apparatus setup, including the particle collection apparatus attachment, is shown in Fig. 2. The air from the laboratory (A) is taken in through a fan (B), which was circulated in a High-Efficiency Particulate Air (HEPA) filter (C) to remove any impurities and dust particles and resulting in the introduction of clean air inside the PoD chamber (D). The air velocity was kept at 11.5 m/s, the magnitude obtained from a previous study^31,32^. Before testing, the air cleanliness was rigorously monitored and maintained below 1 #/cm^3^. To obtain the particle number concentration, a TSI® (TSI Incorporated, Shoreview, USA) Optical Particle Sizer Spectrometer (OPS, model 3330) was connected to the enclosed chamber at point F in Fig. 2. The OPS can measure the total particle number concentration in the size range from 0.3 μm to 10 μm, divided into 16 channels, and with a sampling frequency of 1 Hz. The OPS records and measures particle concentration up to 3000 particles/cm^3^, working with a self-controlled sampling flow rate of 1 l/min^32,33^.

To attain proper conformance between the mating surfaces of the pin and disc, before the testing duration, a 30 min long ‘run in’ or ‘bedding procedure’ was conducted for all pairings. After the run-in process, the actual testing duration was for a continuous 90 min. This duration was selected to observe the establishment of a proper friction layer on the mated surfaces^18^. The instantaneous values of friction coefficient (CoF) and total particle concentration during trials were recorded directly from the software attached to the PoD and the OPS apparatus respectively. The specific wear coefficient (pin wear) was obtained by weighing the pins before and after each trial through an analytical balance with a precision of 10^–4^ g, and with the following equation:

$${\text{K}}_{{\text{a}}} = \frac{V}{{\left( {F \times d} \right)}}$$

where:

*V*: wear volume loss; *F*: load applied; *d*: sliding distance (~ 8150 m).

### Characterization of the materials and pin surface

The worn surfaces of the CFM pin and with slag additions, and the collected wear debris morphology and full frame EDXS analysis were procured through SEM (JEOL IT300), attached with an Energy Dispersive X-ray Spectroscopy (EDXS; Bruker) system.

## Results

### Friction and emission curves

**Figure 3:**
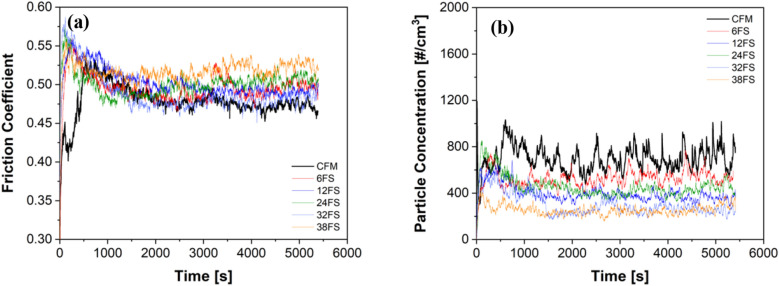
(**a**) Friction traces; (**b**) Emission trends of CFM and with the FS slag additions.

Figure 3a shows the friction trends of CFM and with slag additions. The virgin CFM trends are shown in black. The friction trace achieves a steady state after an initial increase, followed by a gradual decrease and a stabilization of the traces, which is maintained throughout the testing duration. The friction trends of CFM with slag additions observe an increase in the CoF magnitude. Nevertheless, the slag additions do not disturb the steady-state attainment, i.e., also in these cases, the steady-state in the friction traces is achieved like CFM and maintained throughout the length of the testing. Figure 3b shows the emission traces of CFM and with slag additions. The virgin CFM emission trends, shown in black, again achieve a steady state right from the beginning of the testing duration. The fluctuations seen in the friction traces are characteristics of the emission curves. With the slag additions, a decrease in the emission magnitude is observed. Additionally, in all cases of slag additions, the steady state in emission traces is still maintained.

### Comparison of friction, wear, and emission characteristics

**Figure 4:**
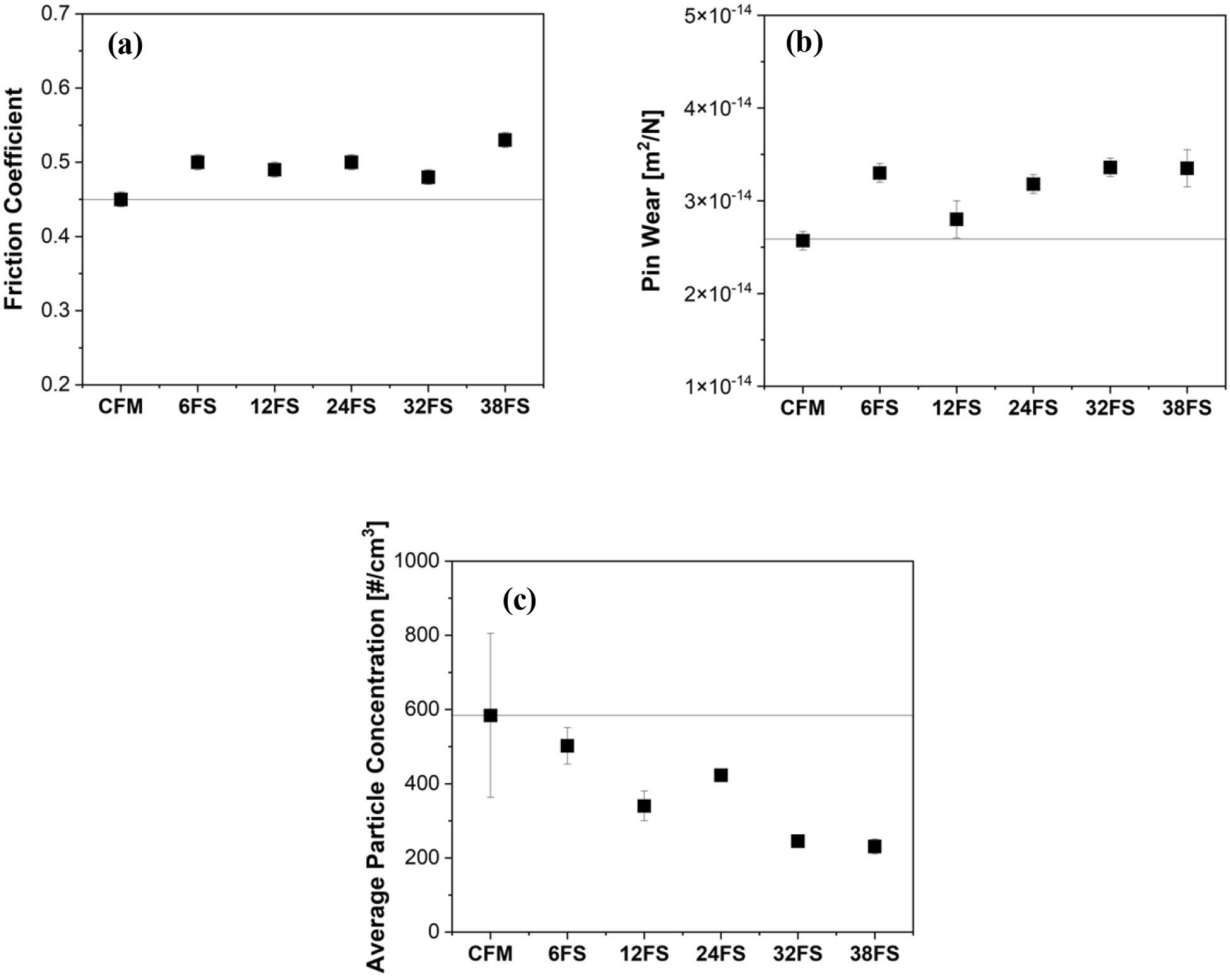
(**a**) Steady state friction coefficient; (**b**) Pin wear; and (**c**) Average particle concentration/average emission magnitude of CFM and with the FS slag additions.

Figure 4 presents the steady state friction coefficient (CoF, Fig. 4a), pin wear (Fig. 4b), and average particle concentration (Fig. 4c) comparison of CFM and with slag additions. In Fig. 4a, an increase in the CoF can be observed with the slag additions, when compared to the virgin CFM. The CoF magnitude increased from 0.45 to around 0.50. The CoF remains similar from 6 wt.% to 32 wt% FS addition but is observed to slightly increase at 38 wt.% addition of FS slag to 0.53. The pin wear in Fig. 4b is observed to increase with the addition of slag content when compared to virgin CFM, tending to an average value around 3.3 × 10^–14^ m^2^/N, which is higher than the wear of the CFM composition but well within the mild wear regime^4,18,31^. Lastly, the average particle concentration of all compositions is depicted in Fig. 4c. The emissions are seen to reduce appreciably with the increase in the slag content. The emission magnitude observes stability in reduction beyond 32 wt% addition of FS slag.

### Worn surfaces and collected wear debris

**Figure 5:**
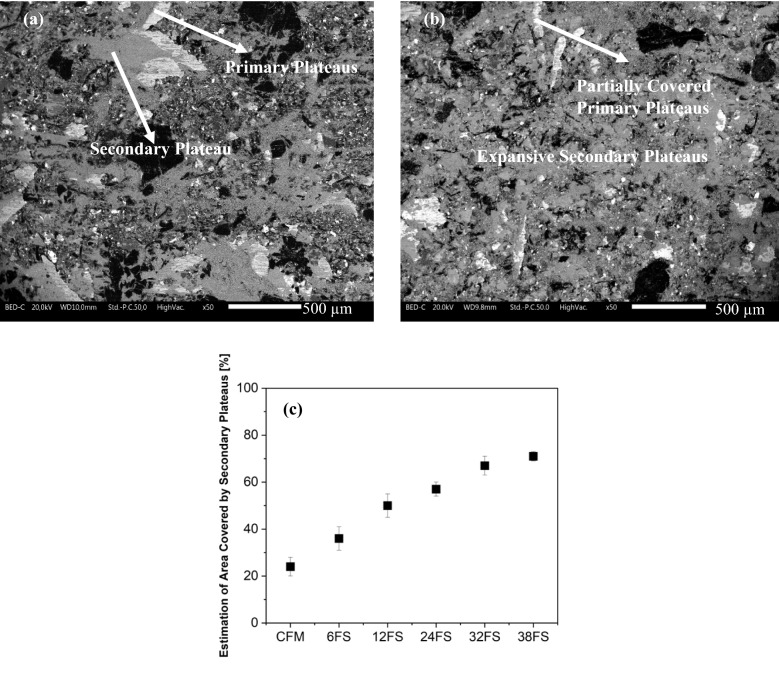
Worn surface characteristics (**a**) CFM; (**b**) CFM + 38FS; (**c**) Estimation of secondary plateau area coverage deposited on CFM and with slag additions.

As an example, Fig. 5a and b show the worn surface characteristics of CFM and CFM with 38 wt% FS respectively. The worn surfaces are typically made of hard reinforcements like steel fibers which act as primary contact plateaus, against which the compacted wear debris, termed secondary contact plateaus, are usually deposited^17,34^. In Fig. 5a, for the worn CFM surface, the white regions are the steel fibers (primary contact plateaus). Against these steel fibers, light grey regions are observed, which are significantly compacted and are the secondary contact plateaus. These plateaus are deposited in the vicinity of the steel fibers and their extension on the surface is quite limited. With the 38 wt% slag addition in Fig. 5b, the extension of the secondary contact plateaus is seen to increase. Another interesting observation is the decrease in the appearance of steel fibers as the slag content is present. This is attributed to a decrease in both the fraction of steel fibers and in their coverage by the extended and compacted secondary plateaus. In fact, with the increase in the slag content, there was an increase in the extension and compactness of secondary contact plateaus. This is shown by a basic estimation of the secondary contact plateau coverage area conducted on the worn pin surfaces using the ImageJ open-source software. Figure 5c presents the area comparison, showing the steady increase in the secondary contact plateau coverage with the increase in the slag content.

**Figure 6:**
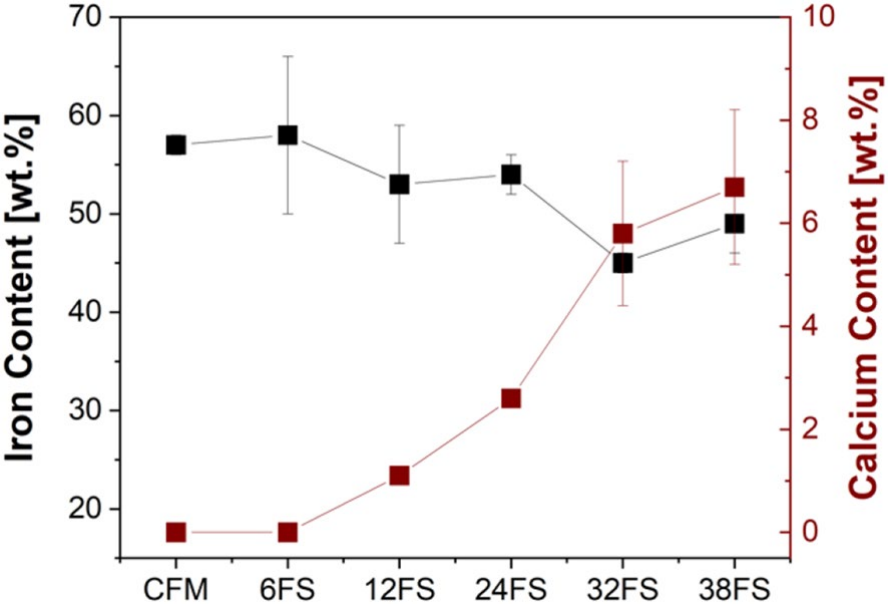
Fe and Ca variation with the increase in the slag content in CFM.

To understand the constituents of the secondary contact plateaus, point/object EDXS analysis was conducted on 8 different secondary plateau sites on multiple specimens for all compositions. Like other previous studies, the secondary contact plateaus were mainly made of Fe and O, symbolizing Fe oxides^32,33,35^. Apart from the Fe oxides, the secondary contact plateaus are made of minor elements like Si, Mg, Al, Mn, Sn, Cr, and Zn. However, for slag containing specimens, the presence of another element was observed, which was Ca. Figure 6 presents the variation in Fe and Ca content concerning CFM and with slag additions. The virgin CFM doesn’t constitute Ca, as seen in the Figure. However, the presence of Ca is seen to increase with the increase in the slag content, symbolizing Ca to be the *marker* element of the FS slag. With 32 and 38 wt% of FS slag addition, a decrease in the Fe content was observed, which could be attributed to the increase in the Ca content, denoting a significant slag contribution to the formation and sustenance of the secondary contact plateaus.

**Figure 7:**
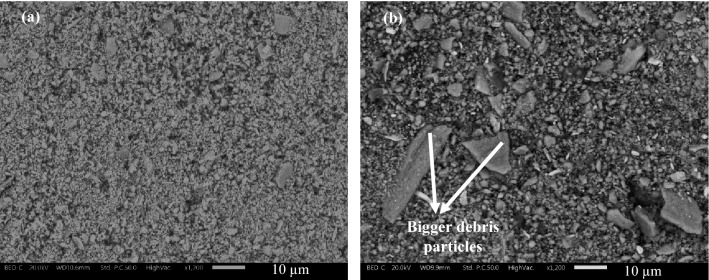
Wear debris morphology (**a**) CFM; (**b**) CFM with 38wt% of FS.

Lastly, the collected wear debris deposited on the disc holder of the PoD equipment was subjected to SEM/EDXS analysis. As an example, Fig. 7a and b show the morphology of the debris collected after tests with virgin CFM and CFM with 38 wt% FS respectively. When the Figures are compared, the particles with the virgin CFM are quite small. In contrast, the collected particles with 38 wt% FS is considerably bigger and more numerous when compared to virgin CFM. In both cases, from full-frame EDXS analysis (Table 3), the major constituent of the debris were Fe and O (Fe oxides), inferring the detachment of secondary contact plateaus. Additionally, with the slag containing specimens, the presence of the marker Ca element was observed, showing the slag contribution.

**Table 3:** EDXS analysis on the composition of collected wear debris.

Element	Mass percentage CFM	Mass percentage CFM with 38 wt.% Slag
Oxygen	29.12 ± 4.4	28.67 ± 3.1
Magnesium	3.56 ± 0.87	2.44 ± 0.53
Aluminum	2.98 ± 0.92	2.12 ± 0.98
Silicon	2.01 ± 0.63	1.83 ± 0.04
Sulfur	1.42 ± 0.08	1.39 ± 0.06
Calcium	0 ± 0	2.84 ± 0.86
Iron	53.34 ± 5.2	55.78 ± 4.8
Zinc	4.51 ± 1.8	3.41 ± 1.2
Tin	3.01 ± 0.91	1.44 ± 0.32

## Discussion

With the introduction of slags, an increase in the CoF was observed (Fig. 4a). As mentioned previously, the slag constituents are typically made of abrasive particles which increase the wear of the disc counterface. The wear particles from the counterface are mainly made of Fe. During sliding, they are then oxidized, intermixed with wear particles originating from the friction material, and finally compacted against the primary plateaus. This induces an increase in the extension of the secondary plateaus (Fig. 5), which is accompanied by an increase in the CoF and the pin wear (Fig. 4b)^18,31^. However, it must be noted that the pin wear with slags is still in the acceptable and same wear regimen as that of the virgin CFM (mild category).

With the increase in the slag content, a pivotal reduction in average particle concentration/emissions was also observed (Fig. 4c). As seen, with an increase in slag content, the extension as well as the compaction of the secondary contact plateau is seen to increase quite significantly. In fact, the largest slag particles acted as small and distributed primary plateaus. On the other hand, the smallest particles were able to enter the secondary plateaus, especially at the highest addition (32 and 38 wt. %), helping in their compaction. It is a well-known observation that most of the wear particles released from the friction material (including the finest airborne particles) are made of detached secondary contact plateaus^32^. With the presence of slags, which led to the higher extension of the secondary contact plateaus, the detached secondary plateaus were also bigger and chunkier, which is shown in Fig. 7b. These larger particles settle on the disc holder and are not airborne, which leads to a significant reduction in emissions. In contrast, the debris morphology of virgin CFM, Fig. 7a, is smaller than the slag specimens, which can be airborne and lead to higher emission recording, despite the lower total wear rate.

To summarize, the addition of metallurgical slag particles to the CFM formulation induced an increase in the extension and compactness of the secondary plateaus that in turn resulted in an increase in the friction coefficient (a positive result) and in wear (remaining mild in nature). At the same time, a large fraction of wear debris was made of big particles that could not become airborne.

The current study was able to show the effective utilization of the abundantly produced BOF slag in a commercially employed friction material formulation. Though positive results with respect to slag additions were showed by Wang et al.^27^ and Erdogan et al.^28^, the emission comparison and debris analysis were not conducted and the friction material formulation utilized were conventional. Through this study, it was seen that the high slag content didn’t hinder the wear and emission characteristics. Furthermore, with the addition of large slag content (38 wt.%), the brake pad manufacturers require significantly reduced portion of the virgin CFM formulation, thereby reducing the cost of raw materials and in turn the manufacturing costs. In 2020, 12 million cars were put on the market in Europe, which, to a first approximation, corresponds to 96 million pads. The weight of the friction material in each pad is approximately 125 g and then app. 4560 tons of metallurgical slag could be re-used each year in Europe (this number is expected to increase further) in place of conventional abrasives (that today have a price of approx. 2 Euros per kilogram). In addition, the European car fleet amounts to approx. 300 million vehicles, and the consumption of friction pads in these vehicles would amount to approx. 300 million pads each year^30^. The production of metallurgical slag is very high (approx. 34 and 16 Mt of blast furnace and steel furnace slags in Europe, respectively) and a considerable fraction is used in the cement industry and civil engineering. However, a large fraction of slag is still landfilled and thus the advantages of beneficially using the slag in friction pads are evident^36,37^. Of course, a specific Life Cycle Assessment within an industrial symbiosis frame is required to evaluate the whole sustainability of the novel friction material from a ‘cradle to the grave’ perspective, considering the actual environmental regulations that limit the landfill disposal of the waste slags.

Finally, the social impact of environmental pollution must be considered. The development of a novel “fully green” friction material, with the engineered re-use of waste materials and a reduction in PM emissions is expected to positively impact the quality of life in Europe, which in the long term will largely contribute to the global sustainable development.

## Conclusion

In this study, a BOF slag was added in a commercially incorporated friction material formulation to check the friction, wear, and emission characteristics at different slag content—6, 12, 24, 32, and 38 wt%.

The CoF increased with the slag addition when compared to the virgin formulation. The highest CoF magnitude was observed for 38 wt% slag content. Like the CoF, the pin wear increased with the slag content addition. However, the pin wear for all compositions were in the mild range. The increase in the CoF and pin wear was attributed to the abrasive nature of the slag particles.

The emissions/average particle concentration steadily decreased with the increase in the slag content. A stability in emission magnitude was seen beyond 32 wt%. The low emissions were due to the presence of smooth, compacted, and extended secondary contact plateaus, the quality of which enhanced with the increase in the slag content. These extended and compacted secondary plateaus produced larger wear debris, which would collect on the disc holder and were not airborne. The presence of slags in the secondary contact plateaus was shown by the marker Ca element, which was not present in the CFM.

These promising results need to be confirmed by specific dynamometric tests, capable of simulating real braking cycles. The future work of this study also includes replacing a few constituents of CFM with slag constituents. Furthermore, the focus will also be provided on a specific Life Cycle Assessment within an industrial symbiosis model, to evaluate the whole sustainability of the novel friction material.

## Data Availability

The data generated during and/or analyzed during the study are available from the corresponding author upon request.

## References

[1] Leonardi M, Menapace C, Matějka V, Gialanella S, Straffelini G (2018). Pin-on-disc investigation on copper-free friction materials dry sliding against cast iron. Tribol. Int..

[2] Federici M, Straffelini G, Gialanella S (2017). Pin-on-disc testing of low-metallic friction material sliding against HVOF coated cast iron: Modelling of the contact temperature evolution. Tribol. Lett..

[3] Chandra P (2015). Braking pad-disc system: Wear mechanisms and formation of wear fragments. Wear.

[4] Jayashree P, Matějka V, Foniok K, Straffelini G (2022). Comparative studies on the dry sliding behavior of a low-metallic friction material with the addition of graphite and exfoliated g-C3N4. Lubricants.

[5] Acerbi F, Sassanelli C, Terzi S, Taisch M (2021). A systematic literature review on data and information required for circular manufacturing strategies adoption. Sustainability.

[6] Acerbi F, Taisch M (2020). A literature review on circular economy adoption in the manufacturing sector. J. Clean. Prod..

[7] Acerbi F, Sassanelli C, Taisch M (2022). A conceptual data model promoting data-driven circular manufacturing. Oper. Manag. Res..

[8] Gregson N, Crang M, Fuller S, Holmes H (2015). Interrogating the circular economy: The moral economy of resource recovery in the EU. Econ. Soc..

[9] Baldassarre B (2019). Industrial symbiosis: Towards a design process for eco-industrial clusters by integrating circular economy and industrial ecology perspectives. J. Clean. Prod..

[10] Domenech T, Bleischwitz R, Doranova A, Panayotopoulos D, Roman L (2019). Mapping industrial symbiosis development in Europe_typologies of networks, characteristics, performance and contribution to the Circular Economy. Resour. Conserv. Recycl..

[11] Grigoratos T, Martini G (2015). Brake wear particle emissions: A review. Environ. Sci. Pollut. Res..

[12] Suleiman A, Tight MR, Quinn AD (2016). Assessment and prediction of the impact of road transport on ambient concentrations of particulate matter PM10. Transp. Res. Part D Transp. Environ..

[13] Kazimirova A (2016). Automotive airborne brake wear debris nanoparticles and cytokinesis-block micronucleus assay in peripheral blood lymphocytes: A pilot study. Environ. Res..

[14] Alemani M, Nosko O, Metinoz I, Olofsson U (2016). A study on emission of airborne wear particles from car brake friction pairs. SAE Int. J. Mater. Manuf..

[15] Naidu TS, Sheridan CM, van Dyk LD (2020). Basic oxygen furnace slag: Review of current and potential uses. Miner. Eng..

[16] Öztürk B, Ztürk SÖ, Adigüzel AA (2013). Effect of type and relative amount of solid lubricants and abrasives on the tribological properties of brake friction materials. Tribol. Trans..

[17] Leonardi M, Alemani M, Straffelini G, Gialanella S (2020). A pin-on-disc study on the dry sliding behavior of a Cu-free friction material containing different types of natural graphite. Wear.

[18] Jayashree P, Straffelini G (2022). The influence of the addition of aluminum anodizing waste on the friction and emission behavior of different kinds of friction material formulations. Tribol. Int..

[19] Nogueira APG (2022). Rice husk as a natural ingredient for brake friction material: A pin-on-disc investigation. Wear.

[20] Gehlen GS (2022). Tribological performance of eco-friendly friction materials with rice husk. Wear.

[21] Ikpambese KK, Gundu DT, Tuleun LT (2016). Evaluation of palm kernel fibers (PKFs) for production of asbestos-free automotive brake pads. J. King Saud Univ. Eng. Sci..

[22] Ibrahim RA (2015). Tribological performance of polyester composites reinforced by agricultural wastes. Tribol. Int..

[23] Gangwar S, Pathak VK (2021). A critical review on tribological properties, thermal behavior, and different applications of industrial waste reinforcement for composites. Proc. Inst. Mech. Eng Part L J. Mater. Des. Appl..

[24] Prasad N (2013). Dry sliding wear behavior of aluminium matrix composite using red mud an industrial waste. Int. Res. J. Pure Appl. Chem..

[25] Mutlu I, Sugözü I, Keskin A (2015). The effects of porosity in friction performance of brake pad using waste tire dust. Polimeros.

[26] Singh T, Patnaik A, Chauhan R (2016). Optimization of tribological properties of cement kiln dust-filled brake pad using grey relation analysis. Mater. Des..

[27] Wang Z (2016). Influence of slag weight fraction on mechanical, thermal and tribological properties of polymer based friction materials. Mater. Des..

[28] Erdoğan A, Gök MS, Koç V, Günen A (2019). Friction and wear behavior of epoxy composite filled with industrial wastes. J. Clean. Prod..

[29] Jabbar FJ (2021). Study the thermal properties of epoxy resin reinforced with calcium oxide fibers. Smart Sci..

[30] Lyu Y, Ma J, Åström AH, Wahlström J, Olofsson U (2020). Recycling of worn out brake pads—Impact on tribology and environment. Sci. Rep..

[31] Jayashree P, Rustighi E, Straffelini G (2022). OPEN A novel study on the reduction of non-exhaust particulate matter emissions through system vibration control. Sci. Rep..

[32] Jayashree P, Sinha A, Gialanella S, Straffelini G (2022). dry sliding behavior and particulate emissions of a SiC-graphite composite friction material paired with HVOF-coated counterface. Atmosphere (Basel).

[33] Nogueira APG, Carlevaris D, Menapace C, Straffelini G (2020). Tribological and emission behavior of novel friction materials. Atmosphere (Basel).

[34] Nogueira APG, Leonardi M, Straffelini G, Gialanella S (2020). Sliding behavior and particle emissions of Cu-free friction materials with different contents of phenolic resin. Tribol. Trans..

[35] Menapace C, Leonardi M, Matějka V, Gialanella S, Straffelini G (2018). Dry sliding behavior and friction layer formation in copper-free barite containing friction materials. Wear.

[36] Shen H, Forssberg E (2003). An overview of recovery of metals from slags. Waste Manag..

[37] Jiao W (2020). Utilization of steel slags to produce thermal conductive asphalt concretes for snow melting pavements. J. Clean. Prod..

